# HSV-1-induced activation of NF-*κ*B protects U937 monocytic cells against both virus replication and apoptosis

**DOI:** 10.1038/cddis.2016.250

**Published:** 2016-09-01

**Authors:** Francesca Marino-Merlo, Emanuela Papaianni, Maria Antonietta Medici, Beatrice Macchi, Sandro Grelli, Claudia Mosca, Christoph Borner, Antonio Mastino

**Affiliations:** 1 Department of Chemical, Biological, Pharmaceutical, and Environmental Sciences, University of Messina, Messina 98166, Italy; 2 Department of Systems Medicine, University of Rome “Tor Vergata”, Rome 00133, Italy; 3 Department of Experimental Medicine and Surgery, University of Rome “Tor Vergata”, Rome 00133, Italy; 4 Institute of Molecular Medicine and Cell Research, Albert Ludwigs University of Freiburg, Stefan Meier Strasse 17, Freiburg D-79104, Germany; 5 Spemann Graduate School of Biology and Medicine (SGBM), Albert Ludwigs University of Freiburg, Albertstrasse 19a, Freiburg D-79104, Germany; 6 BIOSS, Centre for Biological Signaling Studies, Signalhaus, Schänzlestrasse 18, Freiburg D-79104, Germany; 7 The Institute of Translational Pharmacology, CNR, Rome 00133, Italy

## Abstract

The transcription factor nuclear factor-kappa B (NF-*κ*B) is a crucial player of the antiviral innate response. Intriguingly, however, NF-*κ*B activation is assumed to favour herpes simplex virus (HSV) infection rather than restrict it. Apoptosis, a form of innate response to viruses, is completely inhibited by HSV in fully permissive cells, but not in cells incapable to fully sustain HSV replication, such as immunocompetent cells. To resolve the intricate interplay among NF-*κ*B signalling, apoptosis and permissiveness to HSV-1 in monocytic cells, we utilized U937 monocytic cells in which NF-*κ*B activation was inhibited by expressing a dominant-negative I*κ*B*α*. Surprisingly, viral production was increased in monocytic cells in which NF-*κ*B was inhibited. Moreover, inhibition of NF-*κ*B led to increased apoptosis following HSV-1 infection, associated with lysosomal membrane permeabilization. High expression of late viral proteins and induction of apoptosis occurred in distinct cells. Transcriptional analysis of known innate response genes by real-time quantitative reverse transcription-PCR excluded a contribution of the assayed genes to the observed phenomena. Thus, in monocytic cells NF-*κ*B activation simultaneously serves as an innate process to restrict viral replication as well as a mechanism to limit the damage of an excessive apoptotic response to HSV-1 infection. This finding may clarify mechanisms controlling HSV-1 infection in monocytic cells.

The transcription factor nuclear factor-kappa B (NF-*κ*B) plays a fundamental role in the innate antiviral response, acting as a hub network in signal transduction that connects cellular sensors of viral invasion with effector mechanisms of antiviral defence.^1, 2^ Herpes simplex viruses 1 and 2 (HSV-1 and HSV-2) are two closely related large DNA viruses that can infect a variety of cell types, using insidious strategies to modify the molecular organization of the host cell to their own advantage.^3^ HSV-1 can activate NF-*κ*B.^4^ Right after infection, NF-*κ*B activation is independent on virus entry or viral protein expression, as we first demonstrated,^5^ and is triggered by viral structural proteins, such as gD and gH/gL, or the UL37 tegument protein and their interactions with specific receptors.^5, 6, 7, 8, 9, 10, 11^ Later, beginning at 3–6 h post infection (p.i.), NF-*κ*B activation requires the *de-novo* expression of viral proteins.^12, 13, 14^ Noticeably, NF-*κ*B activating components in HSV-1 have not been related with an antiviral role of NF-*κ*B signalling, but rather with a subtle strategy of virus invasiveness.^15, 16^ Various studies indeed suggested that inhibition of NF-*κ*B activation decreased the efficiency of HSV-1 replication in permissive cells.^15, 17, 18, 19^ However, all data showing a pro-virus connotation for NF-*κ*B activation during HSV-1 infection were obtained in fully permissive cell lines. No data are actually available for functions exerted by NF-*κ*B with regard to HSV-1 infection of monocytic cells.

Apoptosis is another evolutionary conserved form of innate response to virus infection and viruses belonging to different families evolved efficient strategies to counteract it.^20, 21, 22^ Actually, numerous proteins of HSVs capable of preventing apoptosis during infection have been identified.^23^ Nevertheless, although inhibition of apoptosis is the prevalent outcome of HSV infection in permissive cells, monocytic and dendritic cells as well as lymphocytes have been shown to undergo apoptosis after infection by HSV.^24, 25, 26, 27, 28, 29, 30^ All these cell types share the common characteristic of undergoing low-productive or abortive infections following exposure to HSV, for reasons that have not been completely elucidated. Interestingly, we have recently demonstrated that apoptosis by HSV-1 in monocytic cells depends on Bax/Bak and the BH3-only Puma protein.^31^ In addition, Bcl-2 overexpression in U937 monocytic cells infected with HSV-1^31^ or HSV-2^32^ leads to an increase of virus yield, further supporting the notion that virus replication in this cell type can be strongly affected by modulating the apoptotic pathway.

Interestingly, a complex interplay exists between NF-*κ*B signalling and apoptosis. In fact, NF-*κ*B was shown to promote both inhibition and induction of apoptotic cell death depending on the context.^33^ During HSV infection, NF-*κ*B translocation to the nucleus has been associated with apoptosis prevention in permissive cells.^34, 35^ Contrasting results, however, were obtained by another group.^36^ With regard to monocytic cells, we demonstrated that activation of NF-*κ*B following infection with HSV-1 as well as upon exposure to HSV-1 glycoprotein D prevented Fas- or staurosporine-induced apoptosis, and this effect was associated with an increased expression of NF-*κ*B-dependent anti-apoptotic genes such as c-IAP2, FLIP and survivin.^5, 37^ Furthermore, we recently reported that cells that expressed a dominant negative (DN) form of I*κ*Bα displayed high levels of apoptosis when infected with wild type HSV-1.^31^ These results support a role for NF-*κ*B activation in preventing apoptosis during HSV infection that can vary with respect to the context and the ability of the cells to sustain virus replication. Thus, resolving the intricate interplay between NF-*κ*B signalling, apoptosis and HSV-1 replication remains an intriguing issue to better understand the complex processes controlling HSV infectious cycle and the restriction of infection in some cell types.

The primary objective of this study was to investigate the relationships among NF-*κ*B activation, viral replication and apoptosis in monocytic cells infected by HSV-1. For this purpose, we used U937 monocytic cells, known to sustain a restricted virus replication cycle and to be susceptible to apoptosis following HSV-1 infection.

## Results

### HSV-1 infection triggers NF-*κ*B activation in control U937 transfectants but not in U937 cells stably transfected with a DN murine I*κ*B

**Figure 1:**
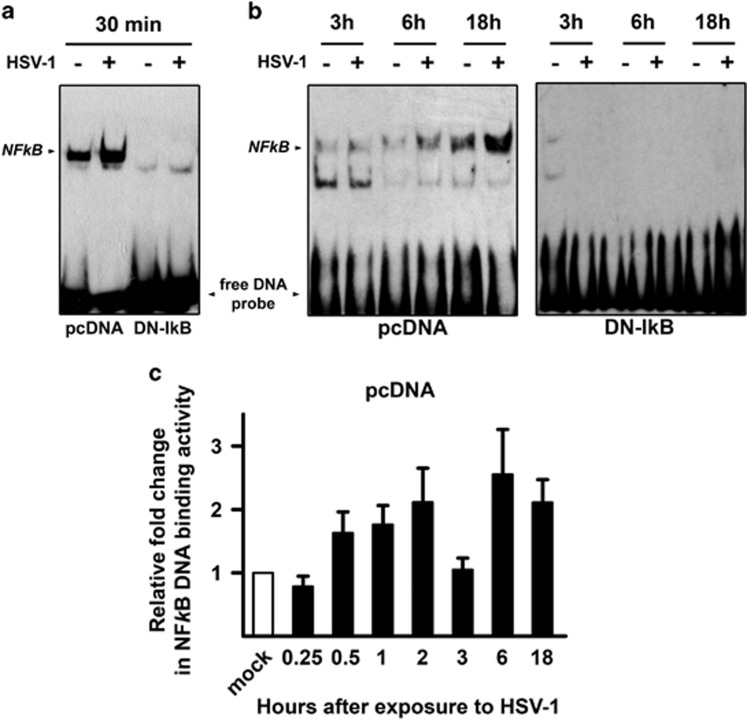
NF-*κ*B DNA binding activity by EMSA in U937 monocytic cells infected by HSV-1. U937 cells stably transfected with an empty control vector (pcDNA) or with a dominant negative (DN) mutant of murine I*κ*B*α* (DN-IkB), were mock infected (−) or infected (+) with 50 MOIs of HSV-1. At 30 min or 3, 6 and 18 h after the first exposure to virus, nuclear extracts were prepared and NF-*κ*B activation was measured by EMSA. pcDNA and DN-IkB samples collected at 30 min were run in the same gel **(a),** while pcDNA and DN-IkB samples collected at the other time points were run in separate gels **(b).** The position of the NF-*κ*B DNA is indicated. EMSA gels from one representative experiment of the three performed are shown. **(c)** Quantitative analysis of the data shown in (a) and (b) using densitometry image analysis of the EMSA gels. Results are expressed as fold change in band intensities from HSV-1- versus mock-infected samples at the same time point. Data represent the mean values±s.d. of three separate experiments for each time course. Multiple comparisons by Bonferroni's post-hoc ANOVA test gave the following results for time 0.25 h versus other times: versus 1 h, *P*=0.044; versus 2 h and versus 18 h, *P*=0.002; versus 6 h, *P*<0.001; versus all other times, not significant. No appreciable optical densitometry value was detected in EMSA gels from parallel time course experiments in U937-DN-I*κ*B cells (data not shown)

To investigate the effects of NF-*κ*B activation on HSV-1 replication and apoptosis in monocytic cells, we utilized U937 cells in which NF-*κ*B was inhibited by stably expressing a DN form of murine I*κ*B*α* (U937-DN-I*κ*B) and vector control U937 cells (U937-pcDNA).^5^ We first determined the kinetics of NF-*κ*B using electrophoretic mobility shift assays (EMSA). Note that for these experiments the indicated times refer to the first exposure to the virus and not to the end of the adsorption time, as for all other experiments. Figure 1a shows that NF-*κ*B binding activity was increased as early as 30 min after first exposure to HSV-1 at a multiplicity of infection (MOI) of 50 plaque forming units (PFU)/cell in U937-pcDNA as compared to mock-infected cells. Thereafter the binding activity diminished (3 h) but reappeared during a later phase of HSV-1 infection (6–18 h) (Figure 1b). Mock-infected U937-pcDNA showed very low levels of basal NF-*κ*B activation. Complete absence of NF-*κ*B specific bands and accumulation of the free DNA probe at the bottom of the gels in either HSV-1-infected or mock-infected U937-DN-I*κ*B cells indicate that DN-I*κ*B stable transfection rendered U937-DN-I*κ*B cells homogenously refractory to NF-*κ*B activation (Figures 1a and b). Figure 1c shows a comprehensive time course of NF-*κ*B binding activity in U937-pcDNA, based on densitometric ImageJ evaluation of EMSA gels. These data indicate the expected biphasic behaviour of HSV-1-induced NF-*κ*B activation in U937 monocytic cells, as previously reported in different experimental cell systems.^38^ No appreciable optical density was detected in EMSA gels from U937-DN-I*κ*B cells, confirming the reliability of our experimental system (data not shown). Such an optimal inhibition of NF-*κ*B binding activity was not achieved with the IkB*α* phosphorylation inhibitor Bay 11-7085 at concentrations that did not affect the viability in U937 cells (data not shown). We therefore preferred to inhibit NF-*κ*B by DN I*κ*B expression for most of experiments.

### HSV-1 replicates more efficiently in U937 cells with disrupted NF-*κ*B activation

**Figure 2:**
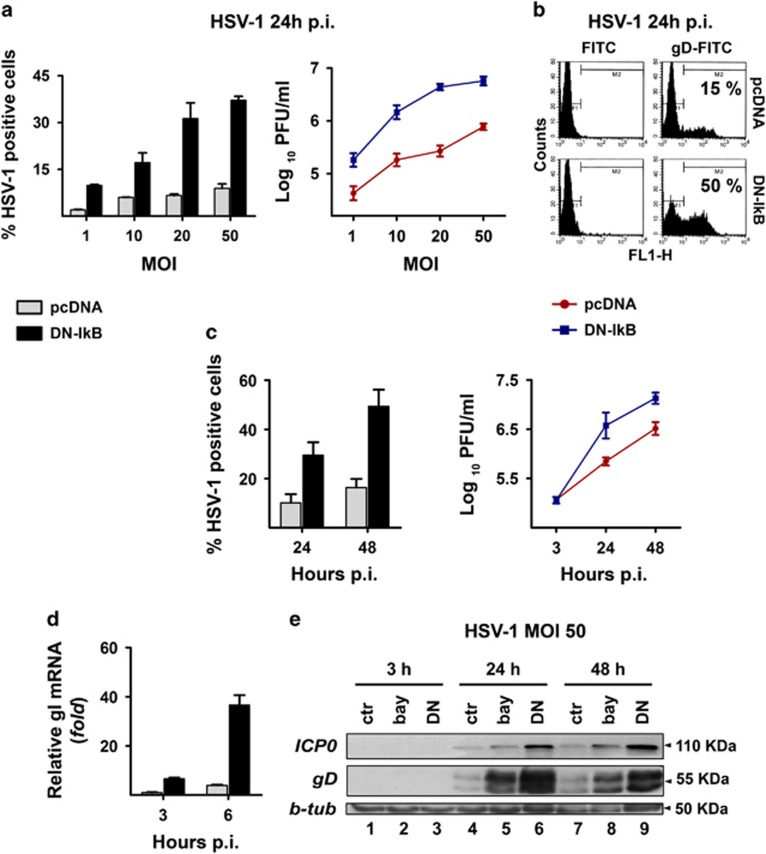
Rate of HSV-1 replication in the presence or in the absence of NF-*κ*B activation in U937 cells. U937-pcDNA and U937-DN-I*κ*B cells were assayed for virus replication by different techniques. **(a)** Cells were infected with different MOIs (1-50) of HSV-1 and analysed at 24 h p.i. by indirect immunofluorescence (left panel) using HSV-1 gD-specific antibody or by plaque-forming assay on Vero cells (right panel). Percentage of gD-HSV-1 positive cells was determined by counting 10 different fields under fluorescence microscope. Viral titres are expressed as Log_10_ PFU/ml in Vero cells of total virus yield from cells infected at the indicated MOI and are depicted in a logarithmic scale (right panel). Data in the left and right panels represent mean values±s.d. from three independent experiments. Left panel, multiple comparisons by Bonferroni's post-hoc ANOVA test gave the following results: pc-DNA versus corresponding DN-IkB groups at all MOI assayed, *P*<0.001; among the pcDNA groups, MOI 1 versus MOI 50, *P*=0.002, all other comparisons, not significant; among the DN-IkB groups, MOI 1 versus MOI 10, *P*=0.001, MOI 20 versus MOI 50, *P*=0.008, all other comparisons, *P*<0.001. Right panel, pcDNA versus corresponding DN-IkB groups at all MOI assayed, *P*<0.001; among the pcDNA groups, MOI 10 versus MOI 20, not significant, MOI 20 versus MOI 50, *P*=0.002, all other comparisons, *P*<0.001; among the DN-IkB groups, MOI 10 versus MOI 20, *P*=0.001, MOI 20 versus MOI 50, not significant, all other comparisons, *P*<0.001. **(b)** Cells were infected with HSV-1 at an MOI of 50 and analysed at 24 h p.i. for viral protein expression by flow cytometry using HSV-1 gD-specific antibody. **(c)** Time dependency of virus replication (MOI 50) assayed as in (a) by immunofluorescence (left panel) or plaque-forming assay (right panel). Data represent the mean values±s.d. from four independent experiments. Left panel, multiple comparisons by Bonferroni's post-hoc ANOVA test gave the following results: pcDNA versus corresponding DN-IkB at all times assayed, *P*<0.001; t24 versus t48, pcDNA not significant, DN-IkB *P*<0.001. Right panel, pcDNA versus corresponding DN-IkB, t3 not significant, all other comparisons, *P*<0.001; among pcDNA and DN-I*κ*B groups at all times assayed, *P*<0.001. **(d)** qPCR analysis of mRNA levels for HSV-1 glycoprotein I in pcDNA cells infected at an MOI of 50 at the indicated times. Data obtained from three independent experiments performed in duplicate were normalized to 18 S rRNA. Values are expressed as mean±s.d. fold changes, relative to gI mRNA levels at 3 h p.i. (the lowest detected value). Multiple comparisons by Bonferroni's post-hoc ANOVA test gave the following results: t6 versus all other groups, *P*<0.001; all other comparisons, not significant. **(e)** Western blot analysis showing the effects of pharmacological (bay lanes 2, 5 and 8) and biochemical (lanes 3, 6 and 9) inhibition of NF-*κ*B activity on expression of IE ICP0 and late gD viral proteins. Whole cell lysates were prepared from HSV-infected (50 MOIs) U937-DN-I*κ*B cells and pcDNA cells, pretreated (bay) or not (ctr) with 1 μM BAY-117085 for 16 h. *β*-tubulin was used as loading control

**Figure 3:**
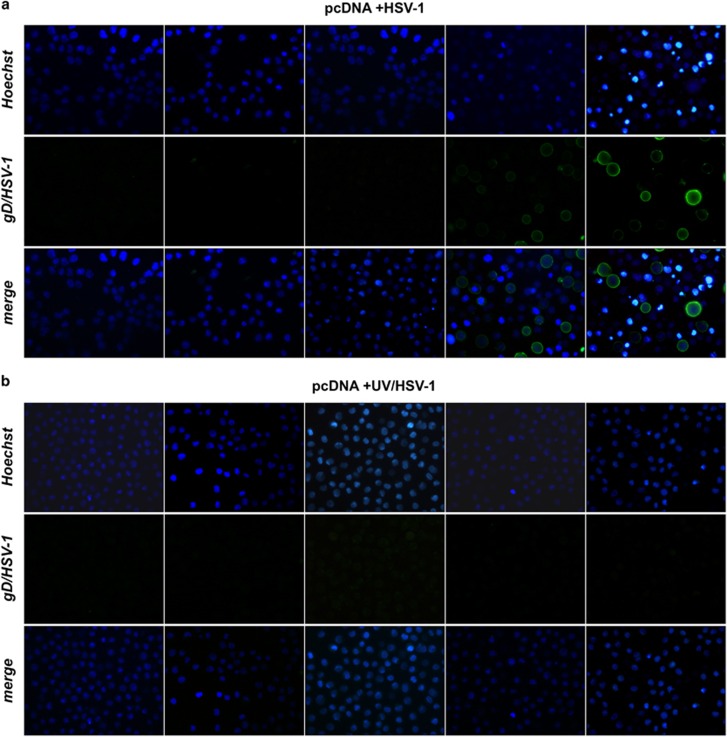

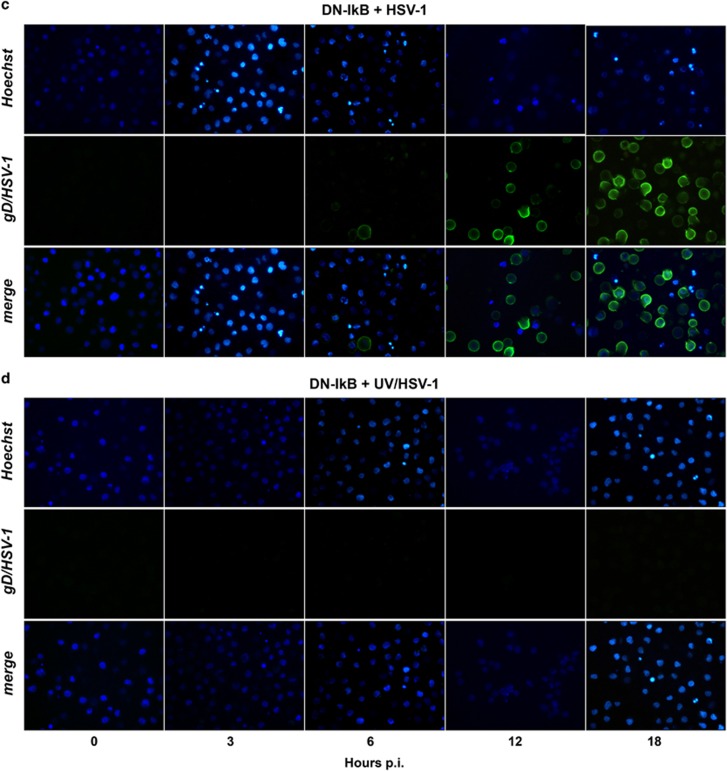
Immunofluorescence microscopy analysis of time-dependent expression of gD-HSV-1 in U937 cells infected with HSV-1 inactivated or not with UV irradiation. **(a)** U937-pcDNA cells were infected with intact HSV-1 (50 PFU/cell). At the end of 1 h adsorption period, the virus inoculum was removed, the cells were washed, fresh medium was added and samples for the first time point were collected and stained (time 0 p.i.). Residual cells were incubated at 37 °C until samples were collected at the indicated hours p.i. Images were captured using fluorescent filters optimized for detecting Hoechst-stained nuclei (upper line), surface gD-expressing cells (middle line) or merged (lower line). **(b)** Parallel infection performed as described in **(a)** on U937-pcDNA cells, but using UV-inactivated HSV-1. **(c)** Parallel infection performed as described in **(a)** but using U937-DN-I*κ*B cells as target cells. **(d)** Parallel infection performed as described in **(b)** but using U937-DN-I*κ*B cells as target cells

Having ascertained that HSV-1 infection triggers NF-*κ*B activation in U937-pcDNA but not in U937-DN-I*κ*B cells viral gene expression and replication were compared between the two cellular systems by various techniques. First, cells were infected at different MOIs and viral gene expression was assessed at 24 h p.i. by immunofluorescence analysis using a HSV-1 gD-specific antibody. U937-pcDNA cells, even at higher MOIs (20–50), were fairly refractory to HSV-1 replication, with only about 10% gD positive cells at 24 h p.i. (Figure 2a, left panel). Conversely, the number of gD-positive cells was remarkably higher in U937-DN-I*κ*B cells and nicely depended on the MOI (Figure 2a, left panel). These results were further confirmed when levels of infective viral particles were determined by titration in the same experiments (Figure 2a, right panel). In addition, the dramatic difference in gD positivity between U937-pcDNA and U937-DN-I*κ*B cells was documented by flow cytometry experiments at 24 h p.i. with HSV-1 (Figure 2b). When we extended the HSV-1 infection time to 48 h, a similar trend was observed. Both the gD positivity and the viral titres were remarkably higher in the U937 cells refractory to NF-*κ*B activation as compared with those in which NF-*κ*B activation was normal (Figure 2c). To get further information on viral replication rate of HSV-1 in monocytic cells with suppressed NF-*κ*B activation, the transcriptional induction of glycoprotein I (gI) of HSV-1 was assessed by real-time RT-PCR analysis during the early phase of infection. As shown in Figure 2d, whereas gI mRNA levels were almost undetectable at 3 h p.i. in U937-pcDNA cells, U937-DN-I*κ*B cells already exhibited a transcriptional induction of gI at the same time that further increased until 6 h p.i. Finally, we monitored changes in the protein levels of the immediate-early protein (ICP0) and of a late protein (gD) by western blot analysis following HSV-1 infection with 50 MOIs in U937-pcDNA and U937-DN-I*κ*B cells as well as in U937-pcDNA cells treated with the NF-*κ*B inhibitor BAY-117085. Again, both the ICP0 and gD protein levels were substantially higher in the cells where NF-*κ*B activation was inhibited after 24 and 48 h p.i. (Figure 2e). This difference was confirmed by densitometric analysis of the bands from three experiments after normalization with beta tubulin (Supplementary Figure 1). No gD positivity was detected in U937-pcDNA and U937-DN-I*κ*B cells infected with UV-irradiated virus at any time assayed (Figures 3b and d), while in cells infected with non-irradiated HSV-1 gD positivity was detected as early as at 6 h after infection in the case of U937-DN-I*κ*B cells and at a lesser extent at 12 h after infection in U937-pcDNA cells (Figures 3a and c), confirming that increased gD positivity was due to *de-novo* transcription and not due to residual gD from virus inoculum. Considering that HSV-1 utilizes the herpesvirus entry mediator (HVEM) receptor to infect monocytic cells, the surface expression of this receptor in U937-DN-I*κ*B and in U937-pcDNA was assessed by flow cytometry analysis, but only a slight difference, too small to explain the remarkable difference in viral replication, was detected between the two cell lines (Supplementary Figure 2). However, to be sure that protection against HSV-1 infection exerted by NF-*κ*B activation did not occur at virus entry level, we titrated the unabsorbed virus in the supernatants collected from cultures of both cell lines infected with HSV-1 at an MOI of 50 PFU/cell at the end of the adsorption period. No difference in residual virus was seen from the supernatants of U937-DN-I*κ*B as compared to U937-pcDNA transfectants (Supplementary Figure 3).

### HSV-1 induces higher levels of apoptosis in U937 cells when NF-*κ*B activation is prevented

**Figure 4:**
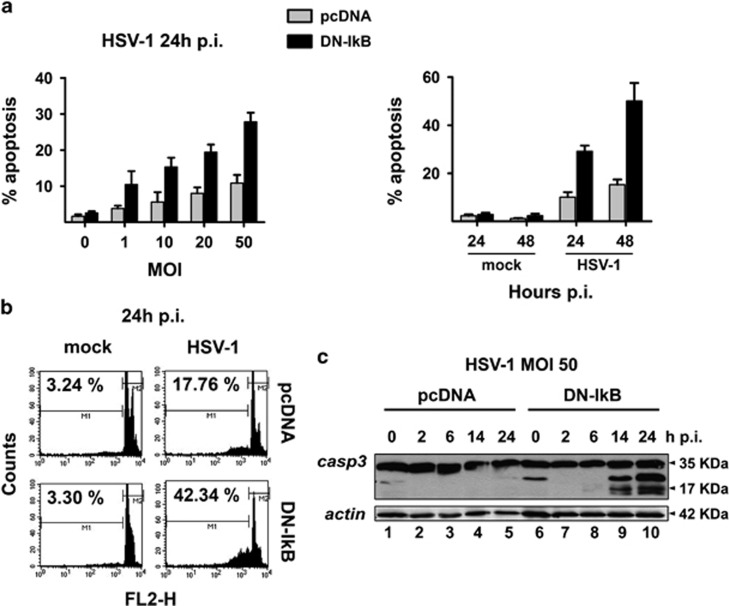
Extent of apoptosis induced by HSV-1 in U937 cells. **(a)** MOI-dependency (left panel) and time-dependency (right panel) of apoptosis as detected by cellular (apoptotic bodies) or nuclear morphology following Hoechst 33342 staining (chromatin condensation, nuclear fragmentation) in HSV-1 infected cells. Apoptotic cells/nuclei were counted in 10 high-power fields for each sample. Data represent mean values±s.d. from three independent experiments. Left panel, multiple comparisons by Bonferroni's post-hoc ANOVA test gave the following results: pc-DNA versus corresponding DN-IkB groups, MOI 0 not significant, MOI 1 *P*=0.002, all other MOI *P*<0.001; among the pcDNA groups, MOI 0 versus MOI 20, *P*=0.003, MOI 0 versus MOI 50, *P*= 0.001, MOI 10 versus MOI 50, *P*=0.024, all other comparisons, not significant; among DN-IkB groups, MOI 1 versus MOI 10, *P*=0.041, MOI 10 versus MOI 20, not significant, all other comparisons, *P*<0.001. Right panel, pc-DNA versus corresponding DN-IkB groups at all times assayed, mock groups not significant, HSV-1 groups *P*<0.001; among the pcDNA groups, mock 24 versus mock 48, not significant, mock 24 versus HSV-1 24, *P*=0.008, mock 24 versus HSV-1 24, mock 24 versus HSV-1 48, *P*<0.001, mock 48 versus HSV-1 24, *P*=0.001, mock 48 versus HSV-1 48, *P*<0.001, HSV-1 24 versus HSV-1 48, not significant; among the DN-I*κ*B groups, mock 24 versus mock 48, not significant, all other comparisons, *P*< 0.001. **(b)** Cell death quantitation by FACS analysis using propidium iodide-stained nuclei. U937-pcDNA and U937-DN-I*κ*B cells were mock-infected or infected with 50 MOI of HSV-1and collected after 24 h for sample preparation. M1 and M2 markers indicate the boundaries between the hypodiploid and the diploid nuclei arbitrarily set on the mock-infected samples and maintained for the infected samples. The numbers represent the percentages of hypodiploid nuclei. **(c)** Western blot analysis of caspase-3 cleavage in HSV-1 infected cells. Whole cell lysates were prepared from both cell lines at the indicated times after infection with 50 MOI HSV-1. Antibodies for procaspase-3 (32 kDa) and the active cleaved form of caspase-3 (20–17 kDa) were used for immunoblot analysis and anti-actin as loading control

The paradigm in virus infection is that levels of virus replication should be inversely correlated with levels of apoptosis, as we previously observed in Bcl-2 overexpressing U937 cells infected with HSV-2^32^ and in a less pronounced manner with HSV-1.^31^ Given that disruption of NF-*κ*B activation enhanced HSV-1 replication in monocytic cells we could expect that levels of apoptosis should be concomitantly diminished. However, NF-*κ*B is considered a survival factor and prevention of its activation should enhance apoptosis.^5^ We, then, determined the extent of apoptosis in U937-pcDNA cells and U937-DN-I*κ*B cells infected with HSV-1 at different MOIs (0–50) at 24 or 48 h p.i. by either counting apoptotic versus intact nuclei under the microscope (Figure 4a) or performing flow cytometry analysis using propidium iodide staining of isolated nuclei (Figure 4b). As shown in Figure 4a, apoptosis was remarkably higher in HSV-1-infected U937-DN-I*κ*B, either at 24 h p.i. or at 48 h p.i., as compared to control U937-pcDNA cells, with a peak of about 50% apoptosis in U937-DN-I*κ*B cells at 48 h p.i with an MOI of 50 PFU/cell. Flow cytometry analysis confirmed that U937 cells with impaired NF-*κ*B activation displayed more apoptotic, sub G1 nuclei following HSV-1 infection at 24 h p.i., than U937 cells with intact NF-*κ*B activation (Figure 4b). Moreover, while caspase-3 was readily cleaved into its active p17 form in HSV-1-infected U937-DN-I*κ*B cells, such a cleavage was hardly seen in the control U937-pcDNA cells (Figure 4c). In addition, exposure to replication-incompetent UV/HSV-1, differently from intact virus, did not give rise to detectable levels of apoptosis, as revealed by Hoechst staining (Figure 3). Overall, these results demonstrate that NF-*κ*B activation restrains the apoptotic response to HSV-1 infection in monocytic cells.

### Higher levels of apoptosis were associated with lysosomal membrane permeabilization (LMP) in U937 cells with impaired NF-*κ*B activation

**Figure 5:**
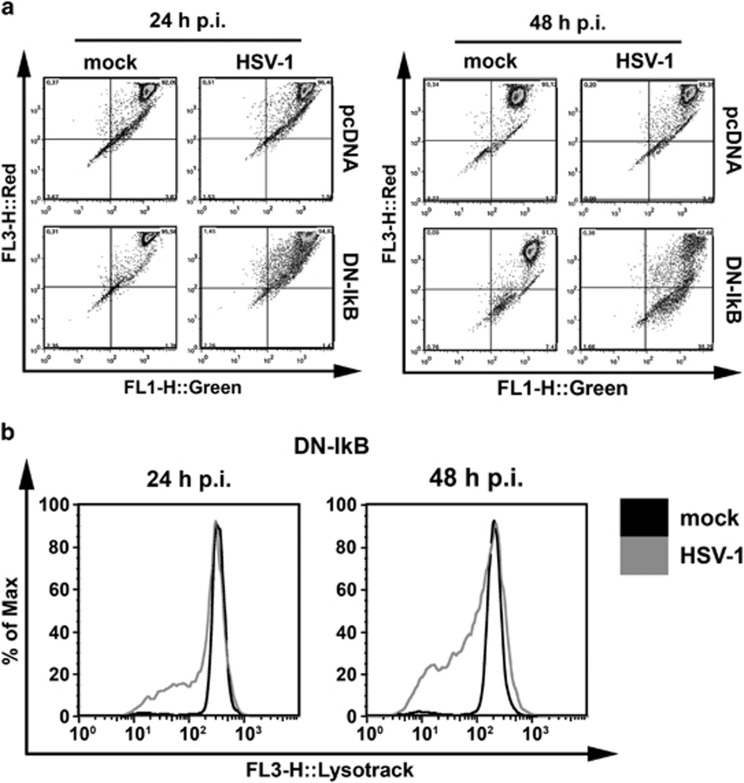
Extent of lysosomal membrane permeability after HSV-1 infection of U937-pcDNA and U937-DN-I*κ*B cells. Quantitative, FACS analysis of mock-infected (mock) or HSV-1-infected (HSV-1) cells (MOI 50 PFU cell^−1^), following staining with 10 μM acridine orange **(a)** or 75 nM LysoTracker Red DND-99 **(b)** at 24 h and 48 h p.i. Dot plots **(a)** and histograms **(b)** show a greater reduction of red fluorescence in the lysosomes (FL3-H) concomitant with an increase in green fluorescence in the cytosol (FL1-H) in HSV-1-infected U937-DN-I*κ*B cells

Lysosomal membrane permeabilization (LMP) and increased cathepsin activities have been recently detected in monocytic cells infected by the apoptosis-inducing d-120-mutant of HSV-1.^39^ To better characterize enhanced levels of cell death induced by wild type HSV-1 in monocytic cells in which NF-*κ*B activation was impaired, we therefore determined the extent of LMP by staining our mock- or HSV-1-infected cells with the lysosomotropic probe acridine orange. At 24 and 48 h p.i. the HSV-1-infected U937-pcDNA cells, which were quite resistant to apoptosis, did not show any reduction of red fluorescence, indicating that LMP did not occur (Figure 5a, left panel). By contrast U937-DN-I*κ*B cells significantly lost their red acridine orange staining with a concomitant increase in green fluorescence at 48 h p.i. (Figure 5a, right panel). Thus, in these cells, apoptosis sensitivity towards HSV-1 was associated with LMP and a possible alteration of lysosomal function. To confirm this finding in U937-DN-I*κ*B cells, the experiment was repeated with the lysosomotropic probe Lyso Tracker Red. Also in this case, we observed a clear reduction of the red fluorescence of Lyso Tracker in HSV-1-infected versus mock-infected U937-DN-I*κ*B cells at 24 and 48 h p.i. (Figure 5b).

### Viral replication and apoptosis preferentially complete their courses in distinct monocytic cells following HSV-1 infection

**Figure 6:**
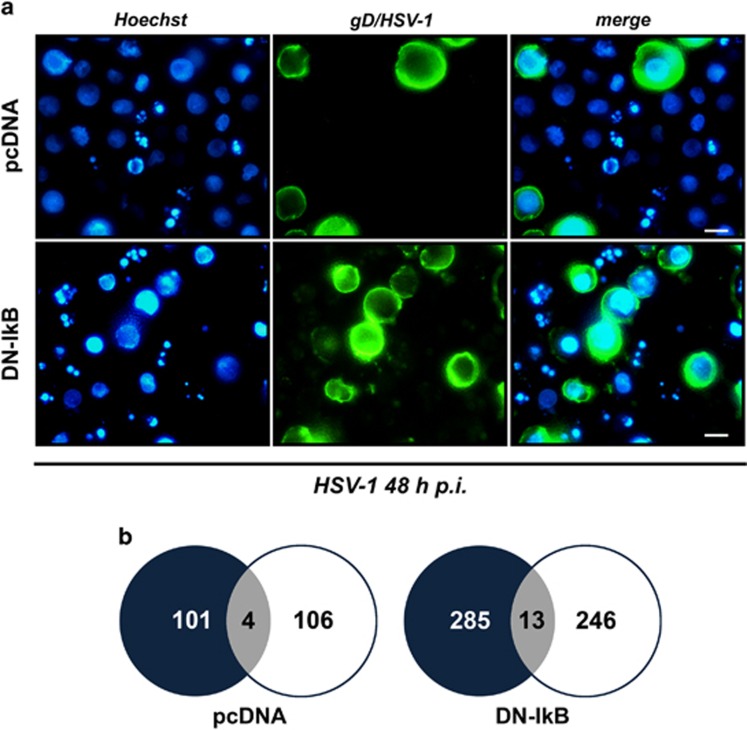
Simultaneous detection of apoptotic and gD/HSV-1-positive cells by immunofluorescence analysis of HSV-1-infected U937-pcDNA and U937-DN-I*κ*B cells. **(a)** Representative microscopy images captured at 48 h p.i., using fluorescent filters optimized for Hoechst-stained nuclei (Hoechst, blue fluorescence, left panel) and surface gD-expressing cells following staining with a mouse anti-gD antibody and an appropriate secondary fluorescein isothiocyanate-conjugated antibody (gD/HSV-1, green fluorescence, middle panel). Images obtained from the same field with different filters were merged (merge, right panel). Fragmented nuclei typical for apoptosis can be observed. Original magnification (630 × ). Scale bar 10 μM. **(b)**Venn diagrams showing the overlap (grey) of apoptotic (black) and gD (white) positive cells for the same fields of a total of 958 counted U937-pcDNA and a total of 949 counted U937-DN-I*κ*B cells, evaluated at 24 h p.i. Data are collected from one representative experiment

Concomitant increase of apoptosis and viral replication in monocytic cells in which NF-*κ*B activation was impeded seemed a paradox. To shed light on this aspect, apoptotic and virus replicative cells were simultaneously detected in U937-pcDNA as well as in U937-DN-I*κ*B cells at the single cell level using fluorescence microscopy analysis. As expected, the percentage of both apoptotic and gD-positive cells were higher under conditions of NF-*κ*B inhibition (Figure 6a, compare upper to lower panels). However, only a few cells stained positive for both apoptosis (nuclear fragmentation) and the late viral gD protein (Figure 6a, right pairs of panels). A similar pattern was observed also in control U937-pcDNA cells showing lower percentages of apoptotic and infected cells. Quantitative data were assembled in Venn diagrams (Figure 6b).

### TNF*α* and IFN*α* do not play a major role in NF-*κ*B-dependant protection against both virus replication and apoptosis in HSV-1-infected monocytic cells

**Figure 7:**
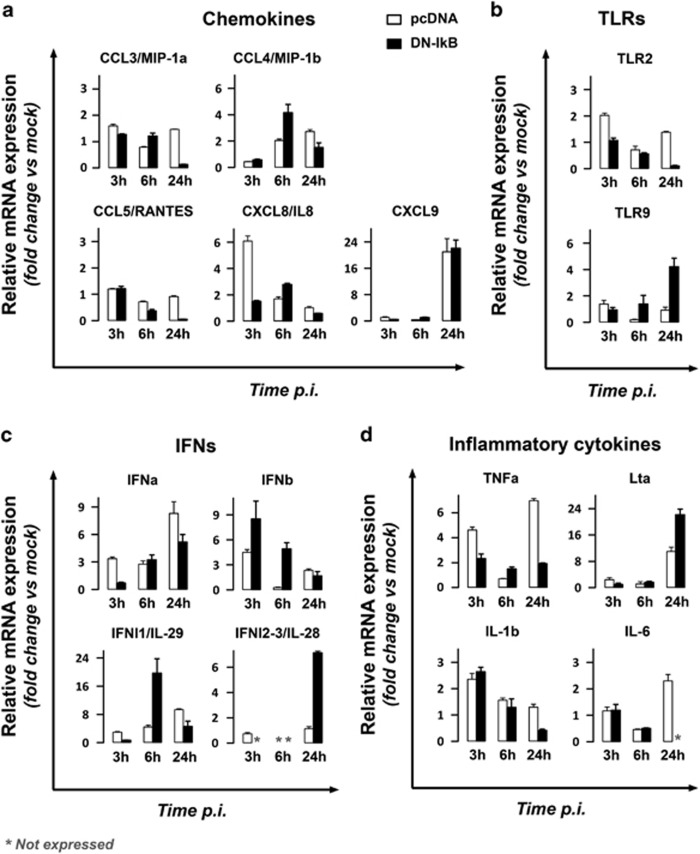
Gene profiling of the antiviral innate response in HSV-1 infected U937-pDNA and U937-DN-I*κ*B using real-time RT-PCR. Total RNA was isolated and reverse transcribed from cells infected with 50 MOI of HSV-1 for 3, 6 and 24 h and respective time-matched mock-infected control cells. Changes in transcripts were quantified by real-time RT-PCR. For each transcript of interest, data were normalized to 18 S rRNA and results were expressed as fold change in mRNA expression of infected cells compared to time-matched mock-infected cells. Bars represent mean values±s.d. from two independent experiments performed in duplicates. Statistical significances of the pcDNA versus the corresponding DN-IkB group, obtained by Bonferroni's post-hoc ANOVA, for each group of genes are the following: **(a)** Chemokines. CCL3: at all times assayed, *P*<0.001. CCL4: t3, not significant; t6, *P*<0.001; t24, *P*=0.005. CCL5: t3, not significant; t6 and t24, *P*<0.001. CXCL8: t3 and t6, *P*<0.001; t24, not significant. CXCL9: at all times assayed, not significant. **(b)**Toll-like-receptors (TLRs). TLR2: t3 and t24, *P*<0.001; t6, not significant. TLR9: t3, not significant; t6, *P*<0.047; t24, *P*<0.001. **(c)** Interferons (IFNs). IFN-alpha (IFNa): t3, *P*<0.006; t6, not significant; t24, *P*=0.001. IFN-beta (IFNb): t3, *P*<0.004; t6, *P*=0.001; t24, not significant. IFN-lambda1 (IFNl1): t3 and t24, not significant; t6, *P*<0.001. IFN-lambda2 (IFNl2): t24, *P*<0.001. **(d)** Pro-inflammatory cytokines. TNF-alpha (TNFa): t3 and t24, *P*<0.001; t6, *P*=0.007. Lymphotoxin alpha (Lta): t3 and t6, not significant; t24,*P*<0.001. IL-1beta (IL-1b): t3 and t6, not significant; t24,*P*=0.001. IL-6: t3 and t6, not significant

In the next step we focused on the cellular events involved on protection exerted by NF-*κ*B activation against virus replication and apoptosis in monocytic cells infected by HSV-1. First, the transcriptional profile of genes known to play a role in the antiviral innate response was compared between U937-pcDNA and U937-DN-I*κ*B cells infected with HSV-1 at 3, 6 and 24 h p.i. in parallel with mock-infected time-matched control cells. The genes were grouped by functional activity and the results were expressed as fold change of mRNA expression calculated by dividing the value obtained from HSV-1-infected cells by that obtained from corresponding mock-infected cells (Figure 7). The selection of possibly implicated genes was based on the following arbitrary criteria: (i) genes showing at least a two-fold change in mRNA expression as compared to mock-infected cells, (ii) genes showing a relative mRNA expression that was significantly higher in U937-pcDNA as compared to U937-DN-I*κ*B cells, at least for two out of the three times assayed. As depicted in Figure 7, none of the genes belonging to the chemokine or Toll-like-receptors (TLR) functional groups satisfied both criteria. Particularly, TLR9 expression was even higher in U937-DN-I*κ*B cells as compared to control cells, suggesting the exclusion of this receptor in NF-*κ*B-dependent restriction of HSV-1 infection in U937 cells. Among the interferon (IFN) and the pro-inflammatory cytokine genes, only IFN*α* and tumor necrosis factor (TNF)-*α* were taken into consideration based on the adopted criteria. In fact, both IFN*α* and TNF*α* were remarkably upregulated in HSV-1 infected U937-pcDNA versus U937-DN-I*κ*B cells at 3 and 24 h p.i.

**Figure 8:**
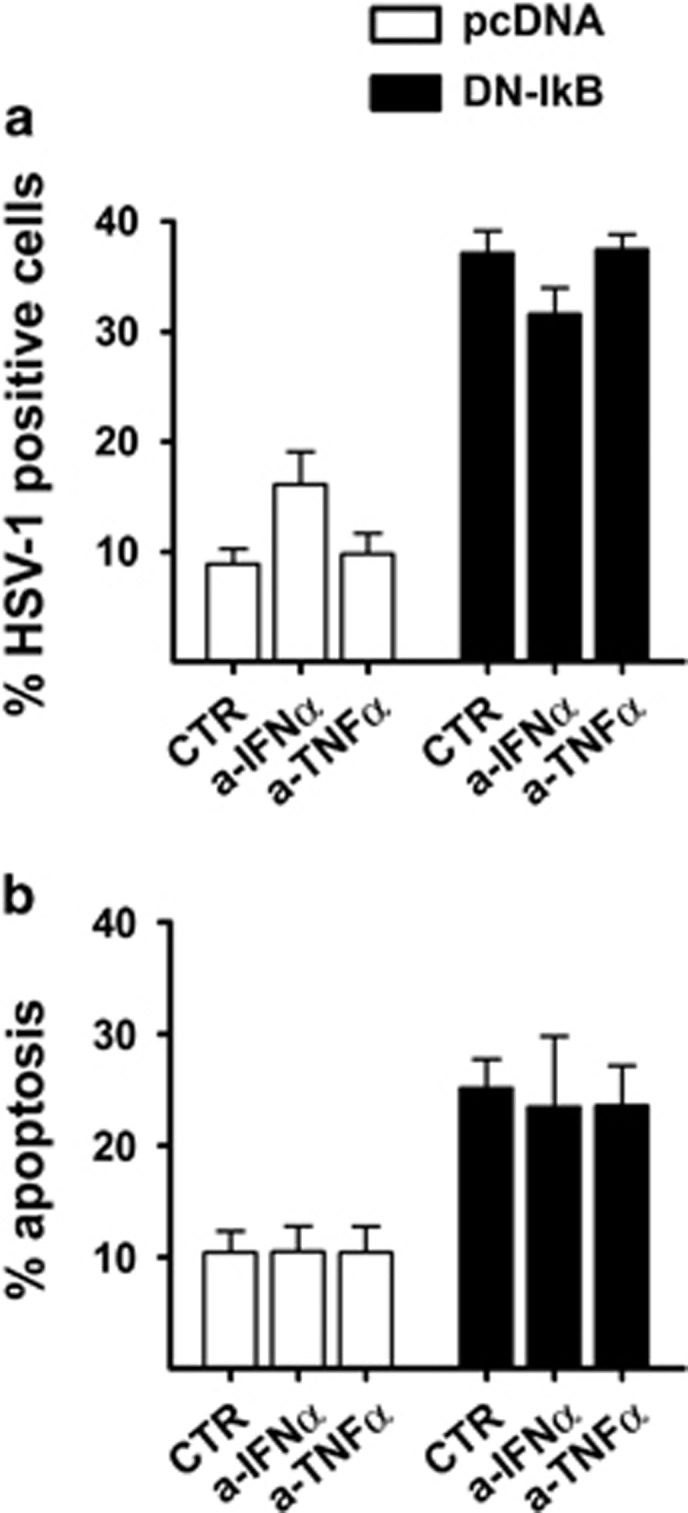
Effects of anti-IFNα and anti-TNFα neutralizing antibodies on the rate of HSV-1 infection and apoptosis. At the end of adsorption time, 10 μg/ml of anti-IFNα (a-IFN*α*) and 20 ng/ml of anti-TNFα (a-TNF*α*), or vehicle (CTR) were added to U937-pcDNA (pcDNA) and U937-DN-I*κ*B (DN-I*κ*B) cultures infected with 50 MOI of HSV-1. After 24 h the samples were collected and analysed by immunofluorescence for gD expression and by PI FACS for apoptosis levels. Bars represent mean values±s.d. from three independent experiments. Results of multiple comparisons by Bonferroni's post-hoc ANOVA test are the following: **(a)** gD/HSV-1 positive cells: among the pcDNA groups, CTR versus a-IFN*α*, *P*=0.017, CTR versus a-TNF*α*, not significant, a-IFN*α* versus a-TNF*α*, *P*=0.044; among the DN-IkB groups, not significant; pcDNA versus corresponding DN-IkB group, *P*<0.001 for all samples. **(b)** Percentage of cells showing nuclear pyknosis (apoptosis): among all pcDNA groups, not significant; among DN-IkB groups, not significant; pcDNA versus corresponding DN-IkB group, CTR, *P*=0.004, a-IFN*α*, *P*=0.010, a-TNF*α*, *P*=0.009

Since both IFN*α* and TNF*α* are well-known NF-*κ*B target genes playing pivotal roles in antiviral innate immunity, in successive experiments neutralizing antibodies directed towards IFN*α* and TNF*α* were applied to HSV-1 infected U937-pcDNA and U937-DN-I*κ*B cell cultures before viral replication and apoptosis were assayed at 24 h p.i. As shown in Figure 8a, the differences between vehicle and anti-IFN*α*-treated U937-pcDNA cell cultures were not statistically significant and no changes were observed in U937-DN-I*κ*B cells (Figure 8a). Thus, as previously seen, gD positivity was largely increased in HSV-1-infected U937-DN-I*κ*B as compared to U937-pcDNA cells but neutralizing antibodies against IFN*α* or TNF*α* had no inhibitory effect on this process. Similar results were obtained when virus titration was utilized for evaluating virus replication (data not shown). Also the extent of apoptosis, which again was higher in the U937-DN-I*κ*B as compared to U937-pcDNA cells, was not affected by neutralizing IFN*α* or TNF*α* during HSV-1 infection (Figure 8b, left graphs).

## Discussion

Previous results indicated that NF-*κ*B activation, rather than playing a role in the host response to virus, increased HSV-1 replication in fully permissive cells.^15, 16, 17, 18, 19^ Recent studies, however, indicate the existence of at least five HSV-1 proteins, including the virion-host shutoff protein, the UL42 DNA polymerase processivity factor, ICP27, ICP0 and the US3 protein kinase, which are capable of antagonizing NF-*κ*B signalling.^40, 41, 42, 43, 44^ This antagonizing function does not fit with an unequivocal pro-virus purpose of NF-*κ*B activation during HSV infection. In fact, from an evolutionary point of view, the presence of multiple anti-NF-*κ*B genes in HSV-1 genome suggests that, at least under particular conditions, NF-*κ*B activation could be detrimental for HSV-1 similar to what occurs in most viral infections. Actually, a study reported an increased HSV-1 replication in permissive cells under conditions in which NF-*κ*B activation was presumably inhibited.^45^ However, this was not found with the wild type virus, but only for the ΔICP0 mutant virus that cannot counteract NF-*κ*B. Nevertheless, this finding suggests the possibility of an antiviral role for NF-*κ*B in HSV infection, at least in particular conditions. Data reported here definitely demonstrate that the facilitating role of NF-*κ*B activation on wt-HSV-1 replication is not an absolute dogma, but depends on the type of the infected host cells. In fact, our results clearly show that in human U937 monocytic cells in which the NF-*κ*B pathway was functionally ablated specifically by stable transfection of a DN i*κ*B*α*, replication of wild type HSV-1 occurred much more efficiently than in NF-*κ*B competent control cells. It is known that not all cell types can fully sustain HSV-1 replication following exposure to the virus. Thus, while cell types fully permissive to HSV-1, such as human epithelial cells, produce a high viral yield, those not completely permissive to HSV-1, including monocytic cells, typically generate only low titres of HSV-1.^46^ Regarding the NF-*κ*B-dependent, down-stream molecular mechanism that restrain virus replication in HSV-1 infected monocytic cells, unfortunately none of the selected, NF-*κ*B-dependent, innate-response-related genes seemed to play a major role. For IFN*α* and TNF*α* we further excluded their major implication. Thus, additional studies are necessary to identify the NF-*κ*B-dependent factor/s responsible for HSV-restriction in the majority of monocytic cells.

Regarding the role of NF-*κ*B signalling for the survival/apoptosis of monocytic cells after HSV-1 infection, one consequence of our results is that restriction of HSV-1 replication in NF-*κ*B-competent monocytic cells is only partially due to levels of apoptosis in these cells: in fact, permissiveness to HSV-1 raises when apoptosis further increases due to NF-*κ*B inhibition. This latter result is not surprising given the fact that NF-*κ*B positively regulates the transcription of anti-apoptotic genes.^33^ Moreover, NF-*κ*B-dependent constriction of the apoptotic response to HSV-1 infection in monocytic cells could be beneficial for the host to limit the damage of an excessive, consequent state of immune suppression. Our data from measuring apoptosis and virus replication at the single cell level allowed us to explain the paradox represented by the simultaneous increase of virus yield and apoptotic cell death in monocytic cells in which NF-*κ*B activation was prevented. In fact, we observed that accomplishment of HSV-1 replication and apoptosis rarely occurred within the same cell, both in NF-*κ*B-incompetent and NF-*κ*B-competent cells. Another novel finding about the process of the apoptotic response to HSV concerns the LMP. In fact, LMP and cathepsins' activities have been previously implicated in apoptosis induced by the d-120-mutant of HSV-1,^39^ but in the present study we clearly demonstrate that induction of LMP is a hallmark of apoptosis induced also by wt HSV-1.

But why are our findings so important for monocytic cells? First, monocytes/macrophages play an important role in the early defence against HSV-1.^47^ Second, HSV-1 diverged from its herpes virus ancestor at the same time as an ancestor of modern humans diverged from a common primate ancestor.^48^ Consequently, long-term coexistence of HSV-1 with its host established a delicate, but extremely efficient balance between the host innate response and the immune evasion strategies of the virus. Our results therefore suggest a scenario in which an efficient NF-*κ*B-dependent innate response to HSV-1 is necessary to limit virus replication as well as an apoptotic response in monocytes/macrophages, thus avoiding an irreparable damage of the cellular innate system of the host that must contribute to locally circumscribe the infection.

In conclusion, results reported in the present study indicate that the fate of HSV-1 infected monocytic cells, whether it is apoptotic cell death/survival or high/low viral replication, depends on a delicate and complex balance between pro-host and pro-virus factors that occur at single cell level and demonstrate that NF-*κ*B signalling plays a fundamental role in controlling it. These results could contribute to the understanding of the still unexplained mechanisms controlling partial restriction of HSV-1 infection that limits permissiveness to the virus of monocytic cells and of the related pathogenetic events.

## Materials and Methods

### Antibodies and reagents

Mouse monoclonal antibodies against HSV-1 gDDL6 (sc-21719), HSV-1 ICP0 (sc-53070), anti- human HVEM ANC3B7 (sc-65284) and anti-*β*-tubulin (sc-55529) were obtained from Santa Cruz Biotechnology (Santa Cruz, CA, USA), mouse anti-human TNF*α* (MAB1021) and mouse anti-human IFN*α* (MAB411) from Chemicon/Millipore (Billerica, MA, USA), rabbit polyclonal antibodies anti-cleaved caspase 3 (#9661) and anti-pro-caspase 3 (#9662) from Cell Signaling Technology (Danvers, MA, USA), and mouse anti-actin monoclonal antibody from MP Biomedicals (Santa Ana, CA, USA). The secondary fluorescein isothiocyanate-conjugated and horseradish peroxidase-conjugated anti-mouse IgG antibodies were obtained from Chemicon/Millipore, the secondary goat anti-mouse IgG phycoerythrin (pe)-conjugated from Santa Cruz Biotechnology. RPMI medium, MEM eagle medium, L-glutamine, penicillin, streptomycin and fetal bovine serum were purchased from Lonza (Basel, Switzerland). All other chemicals and reagents, when not specifically indicated, were purchased from Sigma-Aldrich (St. Louis, MO, USA).

### Cells, virus and treatments

Human monocytic U937 cells and their stable transfectants carrying a DN murine I*κ*Bα (U937-DN-I*κ*B) or a control vector pcDNA3.1 (U937-pcDNA) were grown and cultured as previously described.^5^ HSV-1 ‘F' strain, originally provided by ATCC, was used in all experiments. Virus stocks were produced, titred in Vero cells and stored in aliquots at −80 °C. Monocytic cells were split 24 h before infection and then either mock infected or exposed to different MOIs of HSV-1 for 1 h at 37 °C. For kinetics experiments, cells were exposed to HSV-1 also for a shorter time of 30 min. After the adsorption period the virus inoculum was replaced with RPMI 1640 containing 1% fetal bovine serum and cultures were further incubated at 37 °C for the indicated times (hours p.i.). For UV inactivation, virus suspension was exposed in Petri dish for 150 s to UV light at an intensity of 30 watts from a germicidal lamp situated 10 cm above the sample. For pharmacological I*κ*B*α* phosphorylation inhibition, U937 cells were pre-treated with 1 μM of Bay 11–7085 16 h before HSV-1 infection. The Bay 11-7085 concentration used was chosen on the basis of preliminary experiments performed by trypan blue exclusion to select the non-cytotoxic concentration ranges of the drug on monocytic cells. To neutralize effects of endogenous TNFα and INFα production during HSV-1 infection, cytokine-specific neutralizing antibodies to TNFα and IFNα (Chemicon/Millipore) were added to mock and infected cells at the end of adsorption period. After 24 h of incubation at 37 °C, cells were collected and analysed for gD expression and apoptosis levels.

### Immunofluorescence analysis

U937-pcDNA and U937-DN-mI*κ*B cells, either mock infected or infected with HSV-1, were collected by centrifugation and washed in phosphate-buffered saline (PBS), placed on polylysine*-*coated multiwell slides and fixed for 15 min in PBS containing 3% paraformaldehyde. Cells were then washed twice in PBS and incubated for 1 h at 37 °C with mouse anti-gD DL6 (1:200). After washing twice in PBS, slides were incubated for 45 min at 37 °C with fluorescein isothiocyanate-conjugated goat anti-mouse-IgG secondary antibody in PBS (1:300). For analysis of nuclear morphology, 1 μg/ml of Hoechst 33342 was added to the secondary antibody. Slides were washed in PBS, covered with mounting medium, visualized and photographed by fluorescence microscopy (Leitz, Wetzlar, Germany). For quantitative determinations, images from the same field were taken with green (for fluorescein isothiocyanate-labelled antibody) or blue (for Hoechst-stained nuclei) filters. Ten randomly selected fields (magnification 400 × ; 100 cells per field) were captured for each sample to count gD-positive cells (green filter) or nuclei with apoptotic morphology (blue filter). Merged images were used to simultaneously evaluate double-positive cells and the percentages were determined by counting the total number of nucleated cells in the blue filter. Representative fields were photographed using a 630 × magnification.

For gD detection by flow cytometry, we applied the same protocol of staining used for immunofluorescence microscopy analysis except that Hoechst 33342 was omitted.

### Apoptosis and lysosomal membrane assays

Apoptosis was assessed by microscopy analysis of cellular (apoptotic bodies) or nuclear (chromatin condensation, nuclear fragmentation) morphology following staining with Hoechst 3342 chromatin dye, as previously described by some of us.^25^ In some experiments, apoptosis was also evaluated by flow cytometry analysis of nuclei isolated from the cells following detergent treatment and stained with propidium iodide, using a method that discriminates nuclei from apoptotic, necrotic or viable cells, as previously described.^49, 50^ Samples were run and analysed in a BD FACSCalibur flow cytometer using the CELLQuest II software (BD).

To quantify lysosomal membrane integrity, cells were stained with 10 μM acridine orange for 15 min or with 75 nM LysoTracker Red DND 99 (Invitrogen-Molecular Probes, Paisley, UK) for 45 min at 37 °C. After several PBS washes, the reduction of red or green fluorescence was measured by FACSCalibur.^51^

### Nuclear extracts and electro mobility shift assay (EMSA)

For detecting DNA binding activity of NF-*κ*B present in the nuclei of U937-pcDNA and U937-DN-mI*κ*B cells after HSV-1 infection, non-radioactive EMSA was performed. Nuclear extract preparation and EMSA were carried out according to an earlier study.^11, 37^ Briefly, 1 × 10^7^cells were washed with cold PBS and suspended in 0.4 ml hypotonic lysis buffer A (10 mMHepes, pH 7.9, 1.5 mM MgCl_2_, 10 mMKCl, 0.5 mMDTT, 0.2 mM PMSF) for 20 min on ice and homogenized by passing through a 25-gauge needle. After centrifugation at 12 000 × g for 40 s, nuclear pellets were resuspended in 20 μl ice-cold buffer B (20 mMHepes, pH 7.9, 25% glycerol, 0.42 M NaCl, 1.5 mM MgCl_2_, 0.2 mM EDTA, 0.5 mM DTT, 0.5 mM PMSF) and supplemented with 1x protease inhibitor cocktail (Roche Applied Science, Indianapolis, IN, USA). Following a 20 min incubation on ice (with recurring mixing), samples were centrifuged at 12 000 × g for 5 min and supernatants containing nuclear extracts were collected, aliquoted and immediately stored at −80 °C. The LightShift chemiluminescent EMSA kit (Pierce, Rockford, IL, USA) was utilized to perform the EMSA binding reactions. Specifically, 12 μg of nuclear extracts were incubated with 10 pmoles of double-stranded biotinylated probe containing the NF-*κ*B consensus site, and with 1 μg/μl of poly-dI-dC, to prevent unspecific reaction, in 1 × binding buffer for 20 min at room temperature. The complexes were resolved on a 5% native polyacrylamide/0.5x TBE gel and transferred to a positive charge nylon membrane (Bio-Rad, Hercules, CA, USA). After blotting, DNA was cross-linked by UV and signals from the biotin-labelled probe were detected using reagents provided in the ‘LightShift chemiluminescent EMSA module' (Pierce, Rockford, IL, USA). The NIH ImageJ software (version 1.46r, Bethesda, MD, USA) was used to evaluate densitometry of scanned films from EMSA. The density value in each signal area was calculated following subtraction of background density value evaluated in an adjacent blank area of the same size. This value represents the optical density of each shift in the films. Relative density was calculated as the ratio between HSV-1 and mock-infected cells.

### Western blot analysis

Pellets from 3 × 10^6^ cells were resuspended in NP-40 lysis buffer (50 mM TrisHCl, ph 8.0, 150 mM NaCl, 1% NP40, and freshly added, 1 mM PMSF, 0.5 mM DTT) completed with 1x protease inhibitor cocktails (Roche Applied Science) and incubated on ice for 20 min. After centrifugation at 12 000 × g for 10 min at 4 °C, the supernatant was collected and protein concentration assessed by ‘DC Protein Assay' (Bio-Rad). Equal amount of protein (60 μg) was separated by 10% SDS-PAGE, transferred to nitrocellulose membrane (Bio-Rad) and visualized by reversible Ponceau Red staining for verifying transfer efficiency. After 1 h at room temperature with blocking buffer (TBS, 0.1% Tween-20 with 5% non-fat dry milk) and several washing, the membranes were incubated overnight at 4 °C with primary antibodies diluted 1:1000 in TBS-Tween containing 5% bovine serum albumin antibody, subsequently washed and then incubated with corresponding horseradish peroxidase-conjugated secondary antibody (dilution 1:2000 in TBS-T 5% non-fat dry milk). Binding of antibodies was detected by chemiluminescence staining using the ECL detection kit (Pierce). The ImageJ software was utilized to evaluate densitometric values of western blots.

### Determination of mRNA expression by real-time PCR

U937-pcDNA and U937-DN-I*κ*B cells were infected with HSV-1 at an MOI of 50 PFU/cell. At 3, 6 and 24 h p.i., HSV-1- and mock-infected cells were harvested and total RNA was isolated using TRIzol reagent (Life Technologies, Rockville, MD, USA), according to the manufacturer's instructions. DNase treatment (Thermo Scientific, Waltham, MA, USA) was carried out to remove possible DNA contamination and real-time RT-PCR analysis was performed as described before.^52^ Basically, first-strand cDNA synthesis using 0.25 μg of total RNA and random primers was carried out with ‘high-capacity cDNA Reverse Transcription Kits' (Applied Biosystems, Carlsbad, CA, USA), according to the supplier's instruction. Equal volumes of cDNA synthesis mixture were used for amplification of transcripts with SYBR green PCR Real Master Mix (Bio-Rad). For each transcript/sample, triplicate reactions were performed on a CFX-96 Real-time system (Bio-Rad) with gene-specific primers. All the primers employed were purchased from Primm (Milan, Italy) and are listed in Table1. Each run was completed with a melting curve analysis of the PCR end products to validate the specificity of amplification. Cycle threshold (Ct) values were exported into Excel worksheets for analysis of relative changes in gene expression using the 2^-ΔΔCt^ method.^53^ All cDNA quantities were normalized to 18 S rRNA quantities^54^ obtained from the same plate and the fold changes of gene transcripts for infected samples were expressed relative to their values obtained from mock samples collected at the same time.

## Figures and Tables

**Table 1:** List of primer sequences used for RT-qPCR analysis

**Target gene**	**Gene bank number**		**Sequence (5′→3′)**
CCL3	NM_002983	Sense	CCATGGCTCTCTGCAACCA
		Antisense	GCGGTCGGCGTGTCA
CCL4	NM_002984	Sense	CCTCATGCTAGTAGCTGCCTTCT
		Antisense	GAGGGTCTGAGCCCATTGGT
CCL5	NM_002985	Sense	AGCCTCTCCCACAGGTACCA
		Antisense	GGCAGTAGCAATGAGGATGACA
CXCL8	NM_000584	Sense	GTGCAGTTTTGCCAAGGAGT
		Antisense	CTCTGCACCCAGTTTTCCTT
CXCL9	NM_002416	Sense	GAGTGCAAGGAACCCCAGTAGT
		Antisense	TTGTAGGTGGATAGTCCCTTGGTT
TLR2	NM_003264	Sense	GCGGGAAGGATTTTGGGTAA
		Antisense	TGGTCTTAAATATGGGAACCTAGGA
TLR9	NM_017442	Sense	CTTCCCTGTAGCTGCTGTCC
		Antisense	CCTGCACCAGGAGAGACAG
IFNA1	NM_024013	Sense	GTGGTGCTCAGCTGCAAGTC
		Antisense	TGTGGGTCTCAGGGAGATCAC
IFNB1	NM_002176	Sense	CATTACCTGAAGGCCAAGGA
		Antisense	CAGCATCTGCTGGTTGAAGA
IFNL1	NM_172140	Sense	CGCCTTGGAAGAGTCACTCA
		Antisense	GAAGCCTCAGGTCCCAATTC
IFNL2	NM_172138	Sense	AGTTCCGGGCCTGTATCCAG
		Antisense	GAGCCGGTACAGCCAATGGT
TNF	NM_000594	Sense	GCAGGTCTACTTTGGGATCATTG
		Antisense	GCGTTTGGGAAGGTTGGA
LTA	NM_000595	Sense	CACCTTCAGCTGCCCAGACT
		Antisense	TGCTGTGGGCAAGATGCA
IL1B	NM_000576	Sense	GCGAATGACAGAGGGTTTCTTAG
		Antisense	CACCTTCAGCTGCCCAGACT
IL6	NM_000600	Sense	TACCCCCAGGAGAAGATTCC
		Antisense	TTTTCTGCCAGTGCCTCTTT
HSV-1 US7 (gI)	AJ626527.1	Sense	CCCACGGTCAGTCTGGTATC
		Antisense	TTTGTGTCCCATGGGGTAGT
RNA18S5	NR_003286.2	Sense	GTAACCCGTTGAACCCCATT
		Antisense	CCATCCAATCGGTAGTAGCG

## Abbreviations

NF-*κ*B: nuclear factor-kappa B
HSV: herpes simplex virus
HSV-1: herpes simplex virus 1
HSV-2: herpes simplex virus 2
gD: glycoprotein D
gH: glycoprotein H
gI: glycoprotein I
gL: glycoprotein L
DN: dominant negative
U937-DN-I*κ*B: U937 cells stably expressing a DN I*κ*B*α*
U937-pcDNA: U937 cells stably expressing a control vector
EMSA: electrophoretic mobility shift assay
PFU: plaque forming units
MOI: multiplicity of infection
p.i.: post infection
LMP: lysosomal membrane permeabilization
AO: acridine orange
FITC: fluorescein isothiocyanate
HRP: horseradish peroxidase
Pe: phycoerythrin
FBS: fetal bovine serum

## References

[bib1] Hiscott J. Convergence of the NF-kappaB and IRF pathways in the regulation of the innate antiviral response. Cytokine Growth Factor Rev 2007; 18: 483–490. 17706453 10.1016/j.cytogfr.2007.06.002

[bib2] Le Negrate G. Viral interference with innate immunity by preventing NF-kappaB activity. Cell Microbiol 2012; 14: 168–181. 22050732 10.1111/j.1462-5822.2011.01720.x

[bib3] Roizman B, Knipe DM, Whitley RJ. Herpes simplex virus. In: Knipe DM, Howley PM, Cohen JI, Griffin DE, Lamb RA, Martin MA et al (eds). Fields Virology, 6th edn. vol. 2. Wolters Kluwer Health/Lippincott Williams & Wilkins: Philadelphia, PA, USA, 2013, pp 1823–1897.

[bib4] Rong BL, Libermann TA, Kogawa K, Ghosh S, Cao LX, Pavanlangston D et al. Hsv-1-inducible proteins bind to Nf-kappa-B-like sites in the Hsv-1 genome. Virology 1992; 189: 750–756. 1322599 10.1016/0042-6822(92)90599-k

[bib5] Medici MA, Sciortino MT, Perri D, Amici C, Avitabile E, Ciotti M et al. Protection by herpes simplex virus glycoprotein D against Fas-mediated apoptosis: role of nuclear factor kappaB. J Biol Chem 2003; 278: 36059–36067. 12844494 10.1074/jbc.M306198200

[bib6] Leoni V, Gianni T, Salvioli S, Campadelli-Fiume G. Herpes simplex virus glycoproteins gH/gL and gB bind Toll-like receptor 2, and soluble gH/gL is sufficient to activate NF-kappaB. J Virol 2012; 86: 6555–6562. 22496225 10.1128/JVI.00295-12 PMC3393584

[bib7] MacLeod IJ, Minson T. Binding of herpes simplex virus type-1 virions leads to the induction of intracellular signalling in the absence of virus entry. PLoS One 2010; 5: e9560. 20221426 10.1371/journal.pone.0009560 PMC2832691

[bib8] Liu X, Fitzgerald K, Kurt-Jones E, Finberg R, Knipe DM. Herpes virus tegument protein activates NF-kappaB signaling through the TRAF6 adaptor protein. Proc Natl Acad Sci USA 2008; 105: 11335–11339. 18682563 10.1073/pnas.0801617105 PMC2516262

[bib9] Gianni T, Leoni V, Campadelli-Fiume G. Type I interferon and NF-kappaB activation elicited by herpes simplex virus gH/gL via alphavbeta3 integrin in epithelial and neuronal cell lines. J Virol 2013; 87: 13911–13916. 24109241 10.1128/JVI.01894-13 PMC3838217

[bib10] Sciortino MT, Medici MA, Marino-Merlo F, Zaccaria D, Giuffre M, Venuti A et al. Signaling pathway used by HSV-1 to induce NF-kappaB activation: possible role of herpes virus entry receptor D. Ann NY Acad Sci 2007; 1096: 89–96. 17405920 10.1196/annals.1397.074

[bib11] Sciortino MT, Medici MA, Marino-Merlo F, Zaccaria D, Giuffre-Cuculletto M, Venuti A et al. Involvement of HVEM receptor in activation of nuclear factor kappaB by herpes simplex virus 1 glycoprotein D. Cell Microbiol 2008; 10: 2297–2311. 18671825 10.1111/j.1462-5822.2008.01212.x

[bib12] Hargett D, Rice S, Bachenheimer SL. Herpes simplex virus type 1 ICP27-dependent activation of NF-kappaB. J Virol 2006; 80: 10565–10578. 16928747 10.1128/JVI.01119-06 PMC1641752

[bib13] Diao L, Zhang B, Fan J, Gao X, Sun S, Yang K et al. Herpes virus proteins ICP0 and BICP0 can activate NF-kappaB by catalyzing IkappaBalpha ubiquitination. Cell Signal 2005; 17: 217–229. 15494213 10.1016/j.cellsig.2004.07.003

[bib14] Roberts KL, Baines JD. UL31 of herpes simplex virus 1 is necessary for optimal NF-kappaB activation and expression of viral gene products. J Virol 2011; 85: 4947–4953. 21389131 10.1128/JVI.00068-11 PMC3126170

[bib15] Patel A, Hanson J, McLean TI, Olgiate J, Hilton M, Miller WE et al. Herpes simplex type 1 induction of persistent NF-kappa B nuclear translocation increases the efficiency of virus replication. Virology 1998; 247: 212–222. 9705914 10.1006/viro.1998.9243

[bib16] Amici C, Rossi A, Costanzo A, Ciafre S, Marinari B, Balsamo M et al. Herpes simplex virus disrupts NF-kappaB regulation by blocking its recruitment on the IkappaBalpha promoter and directing the factor on viral genes. J Biol Chem 2006; 281: 7110–7117. 16407234 10.1074/jbc.M512366200

[bib17] Amici C, Belardo G, Rossi A, Santoro MG. Activation of I kappa b kinase by herpes simplex virus type 1. A novel target for anti-herpetic therapy. J Biol Chem 2001; 276: 28759–28766. 11387335 10.1074/jbc.M103408200

[bib18] Taddeo B, Zhang W, Lakeman F, Roizman B. Cells lacking NF-kappaB or in which NF-kappaB is not activated vary with respect to ability to sustain herpes simplex virus 1 replication and are not susceptible to apoptosis induced by a replication-incompetent mutant virus. J Virol 2004; 78: 11615–11621. 15479802 10.1128/JVI.78.21.11615-11621.2004 PMC523294

[bib19] Gregory D, Hargett D, Holmes D, Money E, Bachenheimer SL. Efficient replication by herpes simplex virus type 1 involves activation of the IkappaB kinase-IkappaB-p65 pathway. J Virol 2004; 78: 13582–13590. 15564469 10.1128/JVI.78.24.13582-13590.2004 PMC533927

[bib20] Galluzzi L, Brenner C, Morselli E, Touat Z, Kroemer G. Viral control of mitochondrial apoptosis. PLoS Pathog 2008; 4: e1000018. 18516228 10.1371/journal.ppat.1000018 PMC2376094

[bib21] Neumann S, El Maadidi S, Faletti L, Haun F, Labib S, Schejtman A et al. How do viruses control mitochondria-mediated apoptosis? Virus Res 2015; 209: 45–55. 25736565 10.1016/j.virusres.2015.02.026 PMC7114537

[bib22] Everett H, McFadden G. Apoptosis: an innate immune response to virus infection. Trends Microbiol 1999; 7: 160–165. 10217831 10.1016/s0966-842x(99)01487-0

[bib23] Goodkin ML, Morton ER, Blaho JA. Herpes simplex virus infection and apoptosis. Int Rev Immunol 2004; 23: 141–172. 14690858 10.1080/08830180490265574

[bib24] Ito M, Koide W, Watanabe M, Kamiya H, Sakurai M. Apoptosis of cord blood T lymphocytes by herpes simplex virus type 1. J Gen Virol 1997; 78: 1971–1975. 9266996 10.1099/0022-1317-78-8-1971

[bib25] Mastino A, Sciortino MT, Medici MA, Perri D, Ammendolia MG, Grelli S et al. Herpes simplex virus 2 causes apoptotic infection in monocytoid cells. Cell Death Differ 1997; 4: 629–638. 14555977 10.1038/sj.cdd.4400289

[bib26] Fleck M, Mountz JD, Hsu HC, Wu J, Edwards CK3rd, Kern ER. Herpes simplex virus type 2 infection induced apoptosis in peritoneal macrophages independent of Fas and tumor necrosis factor-receptor signaling. Viral Immunol 1999; 12: 263–275. 10532654 10.1089/vim.1999.12.263

[bib27] Pollara G, Speidel K, Samady L, Rajpopat M, McGrath Y, Ledermann J et al. Herpes simplex virus infection of dendritic cells: balance among activation, inhibition, and immunity. J Infect Dis 2003; 187: 165–178. 12552441 10.1086/367675

[bib28] Bosnjak L, Miranda-Saksena M, Koelle DM, Boadle RA, Jones CA, Cunningham AL. Herpes simplex virus infection of human dendritic cells induces apoptosis and allows cross-presentation via uninfected dendritic cells. J Immunol 2005; 174: 2220–2227. 15699155 10.4049/jimmunol.174.4.2220

[bib29] Kather A, Raftery MJ, Devi-Rao G, Lippmann J, Giese T, Sandri-Goldin RM et al. Herpes simplex virus type 1 (HSV-1)-induced apoptosis in human dendritic cells as a result of downregulation of cellular FLICE-inhibitory protein and reduced expression of HSV-1 antiapoptotic latency-associated transcript sequences. J Virol 2010; 84: 1034–1046. 19906927 10.1128/JVI.01409-09 PMC2798353

[bib30] Vanden Oever MJ, Han JY. Caspase 9 is essential for herpes simplex virus type 2-induced apoptosis in T cells. J Virol 2010; 84: 3116–3120. 20071584 10.1128/JVI.01726-09 PMC2826057

[bib31] Papaianni E, El Maadidi S, Schejtman A, Neumann S, Maurer U, Marino-Merlo F et al. Phylogenetically distant viruses use the same BH3-only protein puma to trigger bax/bak-dependent apoptosis of infected mouse and human cells. PLoS One 2015; 10: e0126645. 26030884 10.1371/journal.pone.0126645 PMC4452691

[bib32] Sciortino MT, Perri D, Medici MA, Grelli S, Serafino A, Borner C et al. Role of Bcl-2 expression for productive herpes simplex virus 2 replication. Virology 2006; 356: 136–146. 16950491 10.1016/j.virol.2006.08.001

[bib33] Baldwin AS. Regulation of cell death and autophagy by IKK and NF-kappaB: critical mechanisms in immune function and cancer. Immunol Rev 2012; 246: 327–345. 22435564 10.1111/j.1600-065X.2012.01095.x

[bib34] Goodkin ML, Ting AT, Blaho JA. NF-kappaB is required for apoptosis prevention during herpes simplex virus type 1 infection. J Virol 2003; 77: 7261–7280. 12805425 10.1128/JVI.77.13.7261-7280.2003 PMC164802

[bib35] Yedowitz JC, Blaho JA. Herpes simplex virus 2 modulates apoptosis and stimulates NF-kappaB nuclear translocation during infection in human epithelial HEp-2 cells. Virology 2005; 342: 297–310. 16150474 10.1016/j.virol.2005.07.036

[bib36] Taddeo B, Luo TR, Zhang W, Roizman B. Activation of NF-kappaB in cells productively infected with HSV-1 depends on activated protein kinase R and plays no apparent role in blocking apoptosis. Proc Natl Acad Sci USA 2003; 100: 12408–12413. 14530405 10.1073/pnas.2034952100 PMC218771

[bib37] Sciortino MT, Medici MA, Marino-Merlo F, Zaccaria D, Giuffre-Cuculletto M, Venuti A et al. Involvement of gD/HVEM interaction in NF-kB-dependent inhibition of apoptosis by HSV-1 gD. Biochem Pharmacol 2008; 76: 1522–1532. 18723002 10.1016/j.bcp.2008.07.030

[bib38] Santoro MG, Rossi A, Amici C. NF-kappaB and virus infection: who controls whom. EMBO J 2003; 22: 2552–2560. 12773372 10.1093/emboj/cdg267 PMC156764

[bib39] Peri P, Nuutila K, Vuorinen T, Saukko P, Hukkanen V. Cathepsins are involved in virus-induced cell death in ICP4 and Us3 deletion mutant herpes simplex virus type 1-infected monocytic cells. J Gen Virol 2011; 92(Pt 1): 173–180. 20881085 10.1099/vir.0.025080-0

[bib40] van Lint AL, Murawski MR, Goodbody RE, Severa M, Fitzgerald KA, Finberg RW et al. Herpes simplex virus immediate-early ICP0 protein inhibits Toll-like receptor 2-dependent inflammatory responses and NF-kappaB signaling. J Virol 2010; 84: 10802–10811. 20686034 10.1128/JVI.00063-10 PMC2950559

[bib41] Kim JC, Lee SY, Kim SY, Kim JK, Kim HJ, Lee HM et al. HSV-1 ICP27 suppresses NF-kappaB activity by stabilizing IkappaBalpha. FEBS Lett 2008; 582: 2371–2376. 18539148 10.1016/j.febslet.2008.05.044

[bib42] Cotter CR, Kim WK, Nguyen ML, Yount JS, Lopez CB, Blaho JA et al. The virion host shutoff protein of herpes simplex virus 1 blocks the replication-independent activation of NF-kappaB in dendritic cells in the absence of type I interferon signaling. J Virol 2011; 85: 12662–12672. 21937652 10.1128/JVI.05557-11 PMC3209407

[bib43] Zhang J, Wang S, Wang K, Zheng C. Herpes simplex virus 1 DNA polymerase processivity factor UL42 inhibits TNF-alpha-induced NF-kappaB activation by interacting with p65/RelA and p50/NF-kappaB1. Med Microbiol Immunol 2013; 202: 313–325. 23636254 10.1007/s00430-013-0295-0

[bib44] Wang K, Ni L, Wang S, Zheng C. Herpes simplex virus 1 protein kinase US3 hyperphosphorylates p65/RelA and dampens NF-kappaB activation. J Virol 2014; 88: 7941–7951. 24807716 10.1128/JVI.03394-13 PMC4097809

[bib45] Gianni T, Leoni V, Chesnokova LS, Hutt-Fletcher LM, Campadelli-Fiume G. Alphavbeta3-integrin is a major sensor and activator of innate immunity to herpes simplex virus-1. Proc Natl Acad Sci USA 2012; 109: 19792–19797. 23150579 10.1073/pnas.1212597109 PMC3511702

[bib46] Daniels CA, Kleinerman ES, Snyderman R. Abortive and productive infections of human mononuclear phagocytes by type I herpes simplex virus. Am J Pathol 1978; 91: 119–136. 206146 PMC2018167

[bib47] Ellermann-Eriksen S. Macrophages and cytokines in the early defence against herpes simplex virus. Virol J 2005; 2: 59. 16076403 10.1186/1743-422X-2-59 PMC1215526

[bib48] Wertheim JO, Smith MD, Smith DM, Scheffler K, Kosakovsky Pond SL. Evolutionary origins of human herpes simplex viruses 1 and 2. Mol Biol Evol 2014; 31: 2356–2364. 24916030 10.1093/molbev/msu185 PMC4137711

[bib49] Matteucci C, Grelli S, De Smaele E, Fontana C, Mastino A. Identification of nuclei from apoptotic, necrotic, and viable lymphoid cells by using multiparameter flow cytometry. Cytometry 1999; 35: 145–153. 10554170 10.1002/(sici)1097-0320(19990201)35:2<145::aid-cyto6>3.0.co;2-2

[bib50] Matteucci C, Minutolo A, Balestrieri E, Marino-Merlo F, Bramanti P, Garaci E et al. Inhibition of NF-kappaB activation sensitizes U937 cells to 3'-azido-3'-deoxythymidine induced apoptosis. Cell Death Dis 2010; 1: e81. 21368854 10.1038/cddis.2010.58 PMC3035897

[bib51] Oberle C, Huai J, Reinheckel T, Tacke M, Rassner M, Ekert PG et al. Lysosomal membrane permeabilization and cathepsin release is a Bax/Bak-dependent, amplifying event of apoptosis in fibroblasts and monocytes. Cell Death Differ 2010; 17: 1167–1178. 20094062 10.1038/cdd.2009.214

[bib52] Minutolo A, Grelli S, Marino-Merlo F, Cordero FM, Brandi A, Macchi B et al. D(-)lentiginosine-induced apoptosis involves the intrinsic pathway and is p53-independent. Cell Death Dis 2012; 3: e358. 22833097 10.1038/cddis.2012.97 PMC3406596

[bib53] Livak KJ, Schmittgen TD. Analysis of relative gene expression data using real-time quantitative PCR and the 2(-Delta Delta C(T)) method. Methods 2001; 25: 402–408. 11846609 10.1006/meth.2001.1262

[bib54] Nystrom K, Biller M, Grahn A, Lindh M, Larson G, Olofsson S. Real time PCR for monitoring regulation of host gene expression in herpes simplex virus type 1-infected human diploid cells. J Virol Methods 2004; 118: 83–94. 15081603 10.1016/j.jviromet.2004.01.019

