# Supplementary material for: HSV-1-induced activation of NF-κB protects U937 monocytic cells against both virus replication and apoptosis
Source: Cell Death Dis. 2016 Sep 1;7(9):e2354–. doi: 10.1038/cddis.2016.250 (PMC5059854; doi:10.1038/cddis.2016.250)
Supplement: Supplementary Figure 1 [file cddis2016250x1.pdf]

Supplementary Figure 1.

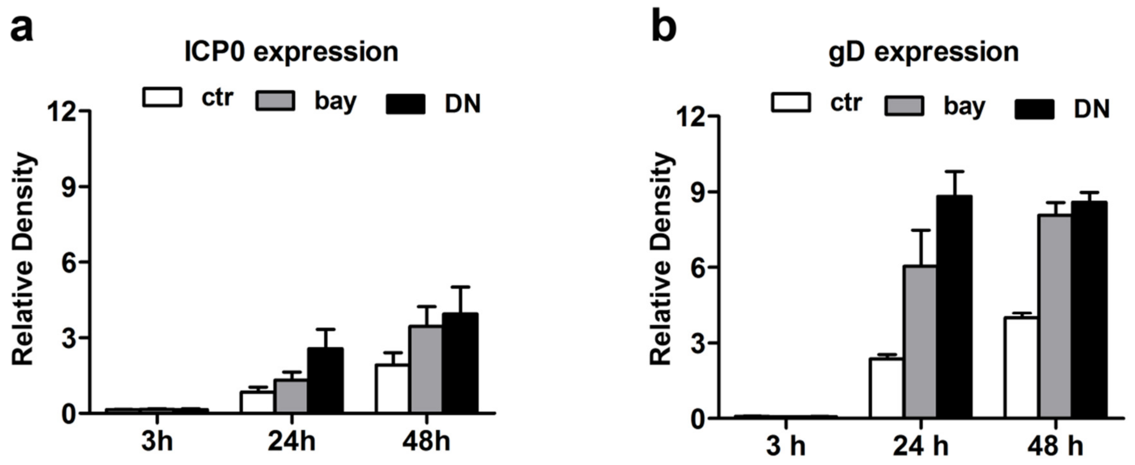

Densitometry analysis of viral protein expression detected by Western blotting in HSV-1 infected cells. U937-pcDNA control cells (ctr), U937-pcDNA cells pretreated with Bay 117085 (bay) and U937-DN-IκB cells (DN) infected with HSV-1 at a MOI of 50/PFU cell, were processed for Western blot analysis of HSV-1 ICP0 (a) and gD (b) viral proteins at the indicated times p.i.. Histograms represent the densitometry analysis of the band intensity from three experiments, performed using ImageJ software. Data are expressed as mean ± S.D. relative density of viral protein/b-tubulin. Comparisons by Bonferroni's post-hoc ANOVA test *versus* infected U937-pcDNA (ctr) group, gave the following results. (a) 24h DN, $P<0.001$; 48h, bay, $P<0.01$, and DN, $P<0.001$. (b) 24h bay and DN, $P<0.001$; 48h, bay and DN, $P<0.001$. A representative experiment is also shown in Figure 2e.
