# Supplementary material for: HSV-1-induced activation of NF-κB protects U937 monocytic cells against both virus replication and apoptosis
Source: Cell Death Dis. 2016 Sep 1;7(9):e2354–. doi: 10.1038/cddis.2016.250 (PMC5059854; doi:10.1038/cddis.2016.250)
Supplement: Supplementary Figure 2 [file cddis2016250x2.pdf]

Supplementary Figure 2.

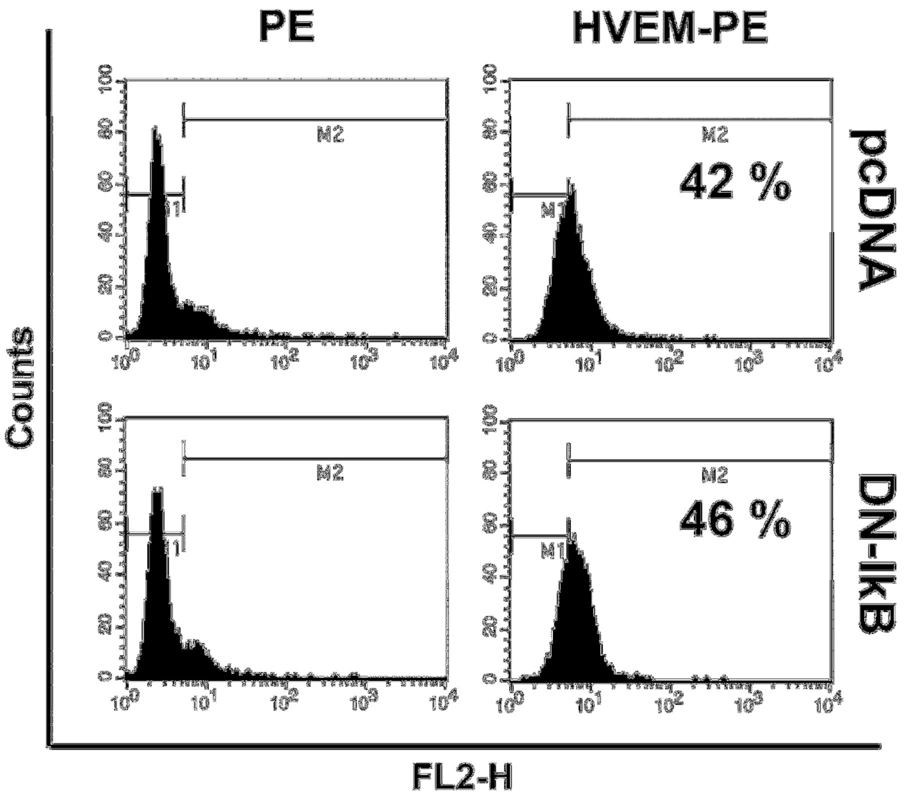

Analysis of HVEM surface expression by flow cytometry. U937-pcDNA (pcDNA) and U937-DN-IκB (DN-IkB) cells were split 24 h before the analysis. Then 1 × 10^6 cells were harvested by centrifugation at 400 x g and washed twice in PBS. After 10 min on ice with blocking buffer (50% FCS and 50% normal goat IgG), cells were incubated with mouse monoclonal anti-HVEM ANC3B7 antibody on ice for 30 min, washed in PBS and incubated with PE-conjugated goat anti-mouse IgG secondary antibody. After 30 min on ice, PBS containing 3% paraformaldehyde was added for a further 15 min on ice, samples were washed and red fluorescence (FL2-H channel) was analysed in a BD FACSCalibur flow cytometer using the CELLQuest II software (BD Biosciences, Franklin Lakes, NJ). Debris and presumably dead cells were excluded from the analysis by adequate gating of scatter parameters. Control samples stained with PE-conjugated secondary antibody only (left panels) were utilized to arbitrarily set the boundary between negative and positive cells. Percentages refer to positive cells after background subtraction of the control sample. One of two replicate experiments is shown.
