# Supplementary material for: HSV-1-induced activation of NF-κB protects U937 monocytic cells against both virus replication and apoptosis
Source: Cell Death Dis. 2016 Sep 1;7(9):e2354–. doi: 10.1038/cddis.2016.250 (PMC5059854; doi:10.1038/cddis.2016.250)
Supplement: Supplementary Figure 3 [file cddis2016250x3.pdf]

Supplementary Figure 3.

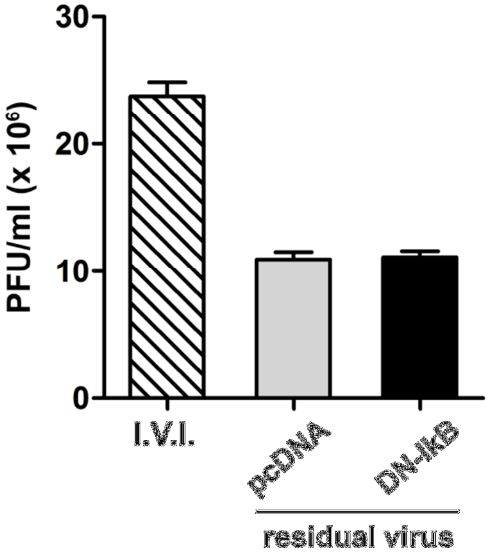

Unadsorbed residual virus in U937-pcDNA and U937-DN-IκB cells. HSV-1 titres were determined in the initial virus inoculum (I.V.I.) and in supernatants collected from cultures of both U937-pcDNA (pcDNA) and U937-DN-IκB (DN-IkB) cell transfectants infected with HSV-1 at a MOI of 50 PFU/cell, at the end of the adsorption period.
